# The Diagnostic Value of Serum Gastrin-17 and Pepsinogen for Gastric Cancer Screening in Eastern China

**DOI:** 10.1155/2021/6894248

**Published:** 2021-04-12

**Authors:** Hongzhang Shen, Kangwei Xiong, Xiangyu Wu, Sile Cheng, Qifeng Lou, Hangbin Jin, Xiaofeng Zhang

**Affiliations:** ^1^Department of Gastroenterology, Affiliated Hangzhou First People's Hospital, Zhejiang University School of Medicine, Hangzhou, China; ^2^Department of Gastroenterology, The Second Hospital of Anhui Medical University, Hefei, China; ^3^Department of Gastroenterology, Huai'an Hospital Affiliated to Xuzhou Medical University and Huai'an Second People's Hospital, China

## Abstract

**Objective:**

To evaluate the diagnostic value of gastrin-17 (G-17) and pepsinogen (PG) in gastric cancer (GC) screening in China, especially eastern China, and to determine the best diagnostic combination and threshold (cutoff values) to screen out patients who need gastroscopy.

**Methods:**

The serum concentrations of G-17 and pepsinogen I and II (PGI and PGII) in 834 patients were analyzed, and the PGI/PGII ratio (PGR) was calculated. According to pathological results, patients can be divided into chronic nonatrophic gastritis (NAG)/chronic atrophic gastritis (CAG)/intraepithelial neoplasia (IN)/GC groups. The differences in G-17, PG, and PGR in each group were analyzed, and their values in GC diagnosis were evaluated separately and in combination.

**Results:**

There were differences in serum G-17, PGII, and PGR among the four groups (NAG/CAG/IN/GC) (*P* ≤ 0.001). In total, 54 GC cases were diagnosed, of which 50% were early GC. There was no significant difference in the PGI levels among the four groups (*P* = 0.377). NAG and CAG composed the chronic gastritis (CG) group. The G-17 and PGII levels in the IN and GC groups were higher than those in the CG group (both *P* ≤ oth *C*), while the PGR levels were lower (*P* ≤ lower). When distinguishing NAG from CAG, the best cutoff value for G-17 was 9.25 pmol/L, PGII was 7.06 *μ*g/L, and PGR was 12.07. When distinguishing CG from IN, the best cutoff value for G-17 was 3.86 pmol/L, PGII was 11.92 *μ*g/L, and PGR was 8.26. When distinguishing CG from GC, the best cutoff value for G-17 was 3.89 pmol/L, PGII was 9.16 *μ*g/L, and PGR was 14.14. The sensitivity, specificity, accuracy, and positive and negative predictive values of G-17/PGII/PGR for GC diagnosis were 83.3%/70.4%/79.6%, 51.8%/56.3%/47.8%, 53.8%/57.2%/49.9%, 10.7%/10.9%/9.6%, and 97.8%/96.5%/97.1%, respectively. The sensitivity, specificity, accuracy, and positive predictive and negative predictive values of PGII/G-17 vs. PGR/G-17 vs. PGR/PGII in the diagnosis of GC were 63.0% vs. 70.4% vs. 64.8%, 70.5% vs. 70.1% vs. 60.4%, 70.0% vs. 70.1% vs. 60.7%, 12.9% vs. 14.0% vs. 10.2%, and 96.5% vs. 97.2% vs. 96.1%, respectively.

**Conclusion:**

The PGII and G-17 levels in patients with gastric IN and GC were significantly increased, while the serum PGR level was significantly decreased. Serological detection is effective for screening GC. The combination of different markers can improve the diagnostic efficiency. The highest diagnostic accuracy was G-17 combined with PGR, and the best cutoff values were G − 17 > 3.89 pmol/L and PGR < 14.14.

## 1. Introduction

Gastric cancer (GC) is the second most common malignant tumor in China. A total of 679,100 Chinese patients were diagnosed with GC in 2015, and 498,000 died as a result. The incidence and death of GC in China accounted for approximately half of the world's total [1]. Many patients have advanced GC when they were first diagnosed, so their prognosis is poor. The 5-year survival rate after surgery is approximately 20-30% [2]. The 5-year survival rate of early GC can reach 90% [2], so early detection and treatment can effectively reduce the mortality rate of GC, but the vast majority of early GC cases are asymptomatic [3]. Therefore, we urgently need a method to improve the detection rate of early GC.

In China, the current national guidelines for early cancer screening suggest screening high-risk groups (refer to materials and methods) from the age of 40. Gastroscopy combined with gastric mucosa biopsy is the gold standard for diagnosing GC. However, gastroscopy and biopsy are invasive operations that may cause some discomfort and complications. Patients do not accept them well. If gastroscopy is performed on the entire high-risk group, it is estimated that only 1-3% of the population would be diagnosed with GC. The efficiency is relatively low, and the examination cost is relatively high, so it is not feasible to conduct gastroscopy surveys in large-scale populations in China [4]. However, if we can use noninvasive methods to screen out patients with a true high risk of developing GC from the high-risk groups and then carry out gastroscopy on these patients, it is expected that the detection rate of early GC can be improved, the screening cost can be reduced, and the compliance of patients can be improved as well.

Anti-Helicobacter pylori IgG antibody (anti-Hp IgG) combined with serum pepsinogen (PG) has been used in large-scale GC screening with satisfactory results in countries such as Japan and Finland. This method is called the “ABC method” [5, 6], but it has some problems in direct application in China. Some studies in China have shown that the overall detection rate of GC in China by the ABC method is low, and its sensitivity and specificity are not as good as those reported abroad [7, 8]. Moreover, China is a country with a high infection rate of Hp. Studies by Shimoyama et al. show that the applicability of PG combined with anti-Hp IgG needs further verification when performing GC screening in areas with a high infection rate of Hp and in elderly populations with a high prevalence rate of atrophic gastritis [9].

Gastrin 17 (G-17) can reflect the atrophy and pathological changes of gastric antrum mucosa. Some scholars proposed using G-17 combined with pepsin as a serological screening indicator [10], which is called the new ABC method in China. Several studies have proven the effectiveness of the new ABC method [7, 8]. However, the types of serological markers and cutoff values for GC screening vary in different regions. In China, studies on the use of the new ABC method to screen GC are still relatively few, and the cutoff values in eastern China have not yet been determined. Therefore, this study aims to explore the effectiveness of serum markers in GC screening in China, especially in eastern China.

## 2. Methods

### 2.1. Patients

The study was conducted on subjects who underwent serological tests and gastroscopy from January 2016 to January 2018 in Hangzhou, Zhejiang Province. All the subjects were aware of the risks and benefits and signed an informed consent form. A total of 834 patients were included in the study. We designed a questionnaire to collect relevant clinical information. The inclusion criteria were as follows: (1) age ≥ 18 years old, regardless of sex. (2) High-risk groups of GC: previous Hp infection; family history of GC; past precancerous lesions such as chronic atrophic gastritis (CAG), gastric polyps, intestinal metaplasia, intraepithelial neoplasia (IN); high-salt diet, smoking, and drinking. The exclusion criteria were as follows: (1) patients with a history of gastric surgery (surgery or endoscopic surgery); (2) patients with esophageal and duodenal diseases; (3) patients administered proton pump inhibitors or acid inhibitors in the past 2 weeks; (4) patients with severe heart, liver, or kidney insufficiencies or mental illness; (5) patients taking anticoagulant drugs such as aspirin and warfarin or those who have blood coagulation dysfunction.

### 2.2. Serological Tests

Five milliliters of venous whole blood was collected from the cubital vein of subjects who fasted for more than 8 hours in the morning. The concentrations of G-17, pepsinogen I (PGI), and pepsinogen (PGII) in serum (E-plate EIKEN H. pylori, Eiken Chemical Co. Ltd, Tokyo, Japan) were quantitatively determined by the enzyme-linked immunosorbent assay (ELISA) method, and the PGI/PGII ratio (PGR) was calculated.

### 2.3. Gastroscopy and Pathological Diagnosis

Gastroscopy was performed by skilled endoscopists (who perform over 1000 endoscopies each year). The gastric mucosa was observed according to standard gastroscopy. Endoscopic diagnosis was based on the Chinese consensus on chronic gastritis (2012, Shanghai) as well as records of other findings. The general analysis of superficial tumors was based on the Paris Classification. All subjects underwent standard biopsies in the antrum and gastric body during gastroscopy, and the biopsy tissue was large and deep enough to reach the muscularis mucosa. If suspicious lesions were found, the biopsy samples were increased according to the size of the lesions. After formalin fixation, dehydration, clearing, paraffin wax immersion, and embedding, sectioning, and staining, the gastric mucosal biopsy tissues were diagnosed by experienced pathologists who did not know the level of the serological markers of the patients in advance. According to the endoscopic and pathological diagnosis, the patients were divided into chronic nonatrophic gastritis (NAG)/CAG/IN/GC/gastric ulcer (GU) groups.

### 2.4. Statistical Analysis

The statistical analysis was carried out by using SPSS statistical software (Version 22.0, SPSS Inc., Chicago, USA). Continuous variables are presented as the mean and standard deviation (*X* ± *S*) and were compared by using *t* test or analysis of variance. Nonparametric tests were used for comparisons between continuous variable groups that do not conform to a normal distribution. Categorical variables are represented by the number of samples and the proportion and were compared by using the *χ*^2^ test or Fisher's exact test. The receiver operating characteristic (ROC) curve and the Youden index (YI) were used to determine the cutoff values. A four-grid table was used to calculate the sensitivity, specificity, accuracy, positive predictive value, and negative predictive value. The difference was statistically significant at *P* < 0.05.

## 3. Results

### 3.1. Baseline Characteristics: Table 1

A total of 834 subjects were included in the study. The mean age of the patients was 54.80 ± 13.195 years, and there were 357 males (42.8%) and 477 females (57.2%).

The diagnoses were as follows: NAG, 346 cases (41.5%); CAG, 332 cases (39.8%); IN, 48 cases (5.8%); GU, 54 cases (6.5%); and GC, 54 cases (6.5%), including 27 cases of early GC (EGC) (3.25%) and 27 cases of advanced GC (3.25%), with the proportion of EGC accounting for 50%.

### 3.2. Differences in G-17, PGI, PGII, and PGR in Different Disease States: Table 2

The laboratory examination results according to different disease states are shown in Table 2. There were significant differences in the serum G-17, PGII, and PGR levels among the four groups (NAG/CAG/IN/GC) (*P* ≤ 0.001). There was no significant difference in the PGI levels among the four groups (*P* = 0.377). NAG and CAG were classified into the chronic gastritis (CG) group. The G-17 levels in the IN and GC groups were higher than those in the CG group (*P* ≤ 0.001). The PGII levels in the IN and GC groups were higher than those in the CG group (*P* ≤ 0.001). The PGR levels in the IN and GC groups were lower than those in the CG group (*P* ≤ 0.001).

### 3.3. Determination of the Cutoff Values of G-17, PGII and PGR and Diagnostic Value: Figure 1

Because there was no difference in the levels of PGI among the groups, we chose G-17, PGII, and PGR as the variables to determine appropriate cutoff values.

**Figure 1:**
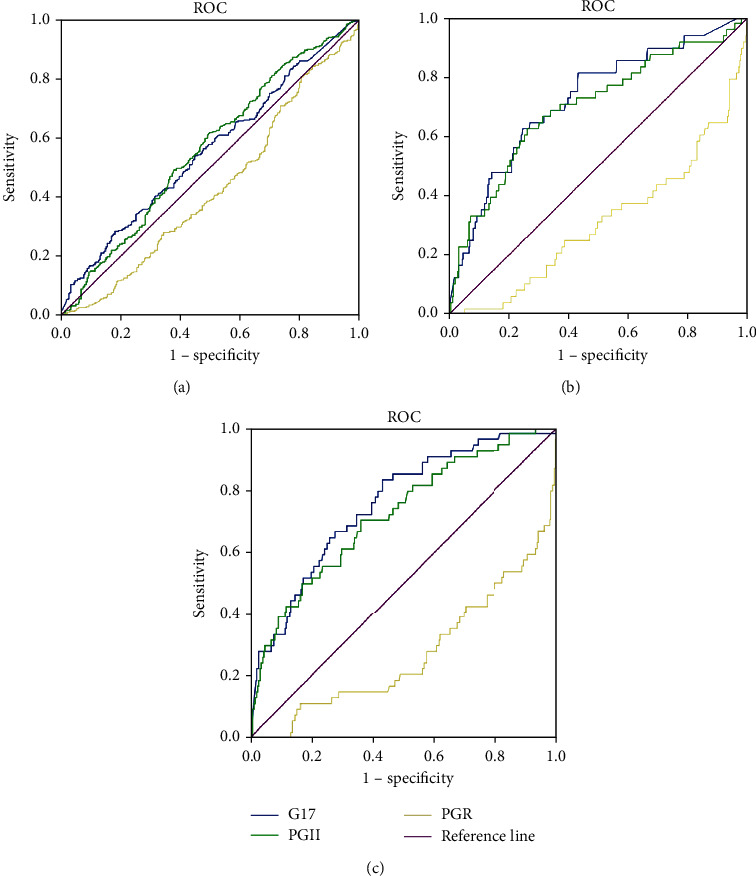
Determination of the cutoff values of G-17, PGII, and PGR and diagnostic value. (a) ROC curve of NAG and CAG. G-17: AUC is 0.555 (CI 0.512-0.598), YI is 0.101, and cutoff value is 9.25; PGII: AUC is 0.561 (CI 0.518-0.604), YI is 0.120, and cutoff value is 7.06; PGR: AUC is 0.571 (CI 0.528-0.614), YI is 0.135, and cutoff value is 12.07. (b) ROC curve of CG and IN. G-17 : AUC is 0.722 (CI 0.642-0.801), YI is 0.379, and cutoff value is 3.86; PGII: AUC is 0.697 (CI 0.611-0.783), YI is 0.362, and cutoff value is 11.92; PGR: AUC is 0.687 (CI 0.604-0.771), YI is 0.328, and cutoff value is 8.26. (c) ROC curve of CG and GC. G-17: AUC is 0.753 (CI 0.684-0.821), YI is 0.403, and cutoff value is 3.89; PGII: AUC is 0.717 (CI 0.642-0.793), YI is 0.342, and cutoff value is 9.16; PGR: AUC is 0.729 (CI 0.650-0.807), YI is 0.357, and cutoff value is 14.14. AUC: area under the receiver operating characteristic curve; YI: Youden index; CI: confidence interval.

When distinguishing NAG from CAG, the best cutoff value of G-17 was 9.25 pmol/L, while that of PGII was 7.06 *μ*g/L and that of PGR was 12.07 (Figure 1(a)). When distinguishing CG from IN, the best cutoff value of G-17 was 3.86 pmol/L, that of PGII was 11.92 *μ*g/L, and that of PGR was 8.26 (Figure 1(b)). When distinguishing CG from GC, the best cutoff value of G-17 was 3.89 pmol/L, that of PGII was 9.16 *μ*g/L, and that of PGR was 14.14 (Figure 1(c)).

### 3.4. Value of G-17 in the Diagnosis of GC

The AUC of G-17 for the diagnosis of GC was 0.753 (95% CI: 0.684-0.821), the maximum value of the YI was 0.403, and the corresponding cutoff value was 3.89 pmol/L. When the diagnostic cutoff value of 3.89 pmol/L was used, the sensitivity, specificity, accuracy, positive predictive value, and negative predictive value of G-17 in the diagnosis of GC were 83.3% (45/54), 51.8% (404/780), 53.8% (449/834), 10.7% (45/421), and 97.8% (404/413), respectively (Table 3).

### 3.5. Value of PGII in the Diagnosis of GC

The AUC of PGII for the diagnosis of GC was 0.717 (95% CI: 0.642-0.793), the maximum value of the YI was 0.342, and the corresponding cutoff value was 9.16 *μ*g/L. When 9.16 *μ*g/L was used as diagnostic cutoff value, the sensitivity, specificity, accuracy, positive predictive value, and negative predictive value of PGII in the diagnosis of GC were 70.4% (38/54), 56.3% (439/780), 57.2% (477/834), 10.0% (38/379), and 96.5% (439/455), respectively (Table 3).

### 3.6. Value of PGR in the Diagnosis of GC

The AUC of PGR for the diagnosis of GC was 0.729 (95% CI: 0.650-0.807), the maximum value of the YI was 0.357, and the corresponding cutoff value was 14.14. When 14.14 was used as the diagnostic cutoff value, the sensitivity, specificity, accuracy, positive predictive value, and negative predictive value of PGR in the diagnosis of GC were 79.6% (43/54), 47.8% (373/780), 49.9% (416/834), 9.6% (43/450), and 97.1% (373/384), respectively (Table 3).

### 3.7. Diagnostic Value of the Combined Use of G-17, PGII and PGR in the Diagnosis of GC

The sensitivity, specificity, accuracy, positive predictive value, and negative predictive value of G-17 combined with PGII (G − 17 > 3.89 pmol/L and PGII > 9.16 *μ*g/L) in the diagnosis of GC were 63.0% (34/54), 70.5% (550/780), 70.0% (584/834), 12.9% (34/264), and 96.5% (550/570), respectively (Table 3).

The sensitivity, specificity, accuracy, positive predictive value, and negative predictive value of G-17 combined with PGR (G − 17 > 3.89 pmol/L and PGR < 14.14 in the diagnosis of GC were 70.4% (38/54), 70.1% (547/780), 70.1% (585/834), 14.0% (38/271), and 97.1% (547/563), respectively (Table 3).

The sensitivity, specificity, accuracy, positive predictive value, and negative predictive value of PGII combined with PGR (PGII > 9.16 *μ*g/L and PGR < 14.14) in the diagnosis of GC were 64.8% (35/54), 60.4% (471/780), 60.7% (506/834), 10.2% (35/344), and 96.1% (471/490), respectively (Table 3).

## 4. Discussion

This study used the so-called new ABC method (based on G-17, PG, and PGR) to screen GC and initially determined the effectiveness of this method and its appropriate threshold in eastern China. According to our results, the use of G-17 combined with PG is effective for screening GC. This combination is also valuable for distinguishing between gastritis and IN.

The serological screening of GC has gradually become increasingly widely used, and there are many different schemes for the serological screening of GC (several of the five markers (PGI, PGII, PGR, G-17, and anti-Hp IgG) have been selected as screening indicators) [5, 11, 12]. However, so far, no scheme is universal. Due to various reasons, the same serological screening program applied to different regions will produce different results [13]. Ghoshal et al. found that PGR and G-17 tests were not good predictors of gastric atrophy and intestinal metaplasia in areas with a low incidence of GC and Hp [13]. In Japan, Finland, and other countries, the effect of using the ABC method to screen GC is better than that in China. In South Korea, Park et al. used the modified ABC method to predict the occurrence of gastric tumors, and using different thresholds can assist in screening gastric adenomas [14]. In Iran, Hosseini et al. used the method of combining PGR, G-17, and anti-Hp IgG to distinguish atrophy from nonatrophy, but the effect was poor [15]. China is a country with a high incidence of Hp infection. The ABC method is not effective, so in this study, we chose the new ABC method proposed in China.

Correa's cascade theory of intestinal-type GC has been widely accepted: GC develops from chronic atrophicgastritis, intestinal metaplasia, and atypical hyperplasia into intestinal-type adenocarcinoma as a multistep and sequential process [16]. Hp infection is an important risk factor for GC and has been widely recognized so far [17–19]. Studies have shown that Hp infection can lead to atrophy and eventually canceration [11, 17, 18], but serological screening methods including Hp antibody in China are not satisfactory [7, 8]. In China, large-scale population studies have shown that there is a weak correlation between Hp infection and GC [20]. It was also mentioned above that when GC screening is performed in areas with a high Hp infection rate and in elderly people with a high prevalence of atrophic gastritis, the applicability of PG combined with the anti-Hp IgG test needs to be further verified [9]. Tu et al. used five indicators, PGI, PGII, PGR, anti-Hp antibody, and G-17, to screen GC, and the effect was better than that of the ABC method [12]. Since the anti-Hp IgG test is of little significance in GC screening in China, a new ABC method based on PG and G-17 was proposed in China. There are a few reports on the screening effect of the new ABC method on GC in China, and the overall reported results are better than those of the ABC method in China [21]. However, the research is still limited, and the threshold (cutoff values) may be different in different regions. Our research used the new ABC method to determine the application effect in eastern China and determine the appropriate threshold.

Gastrin is a gastrointestinal hormone synthesized and released by G cells in the gastric antrum and duodenum. There are many kinds of gastrin molecules in the body; the most important of which are G-17 and gastrin-34 (G-34); G-17 accounts for 80%-90%, and G-34 accounts for 5%-10%. The main function of gastrin is to stimulate parietal cells to secrete hydrochloric acid while regulating the function of the digestive tract to maintain its structural integrity. With the gastric antral mucosa atrophies, the number of G cells decreases, which leads to a decrease in G-17 secretion. With the gastric mucosa atrophies, the number of parietal cells secreting gastric acid decreases, which leads to a decrease in gastric acid secretion. Hypergastrinemia can stimulate cell proliferation and lead to more mutations, which may eventually lead to tumorigenesis. Gastrin receptors are located in both gastric neuroendocrine tumors (NETs) and adenocarcinomas [22]. In addition, it has been reported that GC can simultaneously be found in patients with gastric NETs who are being treated, and patients with atrophic gastritis have been found to have gastric NETs and GC at the same time during follow-up [23]. Hypergastrinemia leads to tumorigenesis not only through the above endocrine pathway but also through local autocrine or paracrine pathways. It has been reported that gastrin peptide and its receptors can be expressed in human gastric adenocarcinoma [24, 25]. In addition, a study has shown that gastrointestinal hormones (such as G-17 and PGII) play a role in the occurrence and development of GC by affecting the inflammatory process and intestinal flora [26]. Our study showed that the serum level of G-17 in GC patients was significantly higher than that of non-GC patients, indicating that individuals with higher serum G-17 levels have a higher risk of developing GC, which is consistent with the results of previous studies [27]. Research conducted by Sun in northeastern China showed that the optimal cutoff value of G-17 for the diagnosis of GC is 10.7 pmol/L, with a sensitivity and specificity of 50% and 83%, respectively [28]. In this study, the optimal cutoff value of G-17 for the diagnosis of GC was 3.89 pmol/L, which is in the upper limit of the normal range. The sensitivity, specificity, accuracy, positive predictive value, and negative predictive value of G-17 for the diagnosis of GC were 83.3%, 51.8%, 53.8%, 10.7%, and 97.8%, respectively. It is clear that the optimal cutoff values in different regions are not exactly the same and are even quite different.

PGs (PGI and PGII) are inactive precursors of pepsin. PGI is secreted by the chief cells and mucous neck cells of the fundus and body. PGII is secreted not only by the chief cells and mucous neck cells of the fundus and body but also by cells in the pyloric gland and Brunner gland. PGI and PGII are secreted into the gastric cavity, with only 1% leaking into circulating blood [29]. When inflammation, atrophy, and intestinal metaplasia occur in the gastric mucosa, the secretion of PG by gastric mucosal cells changes, so the detection of the concentration of PG in serum can reflect the condition of the gastric mucosa [30]. It has been reported that the combined examination of PG and anti-Hp IgG is helpful for discovering the progression of gastric adenoma to Lauren's intestinal-type GC; however, its diagnostic effect for Lauren's diffuse-type GC is poor [31]. In Chile, a study confirmed the strong correlation between PGI and PGI/PGII and gastric precancerous lesions [32]. A case-control study by Cao et al. showed that the serum PGI and PGR levels in the GC group were lower than those in the atrophy group and control group. The diagnostic thresholds of PGI and PGR were 57.15 *μ*g/L and 2.99, respectively; the sensitivity and specificity of the former were 99.3% and 84.5%, respectively, while those of the latter were 92.5% and 89.0% [33]. This study found that there was no significant difference in PGI levels among the groups. The PGII levels in the IN and GC groups were higher than those in the CG group, while the PGR levels in the IN and GC groups were lower than those in the CG group. The optimal cutoff value of PGII for the diagnosis of GC was 9.16 *μ*g/L. With 9.16 *μ*g/L as the diagnostic cutoff value, the sensitivity, specificity, accuracy, positive predictive value, and negative predictive value of PGII for the diagnosis of GC were 70.4%, 56.3%, 57.2%, 10.0%, and 96.5%, respectively. The optimal cutoff value of PGR for the diagnosis of GC was 14.14, and the sensitivity, specificity, accuracy, positive predictive value, and negative predictive value for PGR in the diagnosis of GC were 79.6%, 47.8%, 49.9%, 9.6%, and 97.1%, respectively. PGI had no significant diagnostic value in this study. In contrast to the findings of previous studies, some studies suggest that PGII is a suitable marker for screening gastritis from normal mucosa, but PGI, PGR, G-17, or their combination could not effectively screen patients with precancerous lesions and corpus-predominant gastritis among the first-degree relatives of patients with GC [34]. Some studies also suggest that there is no significant difference in PGI between atrophy and nonatrophy or between GC and non-GC [14, 15]. There are many possible reasons that may be related to region, race, experimental design, incidence of GC, and type of GC [35]. Moreover, these findings show that the detection methods applicable in different areas may not be universal and need to be adapted to local conditions.

Some studies have shown that the diagnostic efficacy of a single serological marker is not good, and the combination of different markers can improve the diagnostic efficiency [7]. Li's study found that the sensitivity, specificity, and diagnostic coincidence rate of G-17 combined with PG in the diagnosis of GC were 62.10%, 75.00%, and 67.86%, respectively [7]. Shiotani's study found that if PGI is combined with G-17 (PGI < 45 ng/mL and G − 17 > 60 pg/mL), the sensitivity and specificity for the diagnosis of GC were 83% and 68%, respectively [36]. The conclusions of this study also suggest that the combination of different markers can indeed improve the diagnostic efficiency. The sensitivity, specificity, accuracy, positive predictive value, and negative predictive value of G-17 combined with PGR (G − 17 > 3.89 pmol/L and PGR < 14.14) in the diagnosis of GC were 70.4%, 70.1%, 70.1%, 14.0%, and 97.2%, respectively. The accuracy of this combination was the highest of all combinations (the accuracies of the other combinations were less than 89.2%).

In addition, it should be noted that there were some patients with GC in this study with no significant changes in the levels of G-17 and PG in their serum. The exclusion of gastric mucosal lesions by serological detection alone may easily cause missed diagnoses in the clinic, so it is necessary to make comprehensive use of all kinds of clinical data in order to obtain the best diagnosis and select the best treatment plan for patients.

## 5. Conclusion

To summarize the above research results, although there are many limitations in this study, we found that the levels of PGII and G-17 in patients with gastric IN and GC were significantly increased, while the level of serum PGR was significantly decreased. Serological detection is effective for screening GC, and combining different markers can improve the diagnostic efficiency, of which G-17 combined with PGR had the highest diagnostic accuracy. The optimal cutoff values are G − 17 > 3.89 pmol/L and PGR < 14.14. Because serological detection is simple and easy, it is suitable for large-scale screening, so it can improve the level of the early diagnosis of GC and reduce the mortality of GC. As the eradication rate of Helicobacter pylori increases, this new ABC method will be more effective for cancer screening in the population. More and more people will benefit from this simple, noninvasive screening.

Of course, due to the different conditions in many regions, more centers and larger populations are still needed to obtain more data.

## Figures and Tables

**Table 1:** Baseline data and disease composition.

Variable	Value
No. of patients	834
Sex	
Male	357 (42.8%)
Female	477 (57.2%)
Mean age ± SD (years)	54.80 ± 13.195
Disease	
Chronic nonatrophic gastritis (NAG)	346 (41.5%)
Chronic atrophic gastritis (CAG)	332 (39.8%)
Gastric ulcer (GU)	54 (6.5%)
Intraepithelial neoplasia (IN)	48 (5.8%)
Gastric cancer (GC)	54 (6.5%)
Early gastric cancer (EGC)	27 (50%)^a^
Advanced gastric cancer	27 (50%)^b^

SD: standard deviation. ^a^Accounts for the proportion of all gastric cancer. ^b^Accounts for the proportion of all gastric cancer.

**Table 2:** Laboratory examination results of different disease states.

	NAG	CAG	IN	GC	P	NAG vs. CAG	CG vs. IN	CG vs. GC
G-17	6.90 ± 9.41	9.56 ± 12.74	16.05 ± 15.03	17.84 ± 16.10	≤0.001	0.002	≤.0.001	≤.0.001
PGI	115.73 ± 51.07	116.31 ± 52.53	127.52 ± 56.46	124.08 ± 62.89	0.377	0.883	0.139	0.279
PGII	10.04 ± 8.42	10.89 ± 7.54	17.22 ± 11.95	18.70 ± 13.74	≤0.001	0.170	≤.0.001	≤.0.001
PGR	15.04 ± 7.28	13.12 ± 6.21	10.36 ± 6.26	9.48 ± 6.54	≤.0.001	≤.0.001	≤.0.001	≤.0.001

**Table 3:** Comparison between the diagnostic value of G-17, PGII, and PGR in gastric cancer and pathological.

	Pathological diagnosis. Total: 834		Sensitivity	Specificity	Accuracy	Positive predictive value	Negative predictive value
	Gastric cancer	Nongastric cancer					
G-17							
Gastric cancer	45	376	83.3%	51.8%	53.8%	10.7%	97.8%
Nongastric cancer	9	404					
PGII							
Gastric cancer	38	341	70.4	56.3%	57.2%	10.0%	96.5%
Nongastric cancer	16	439					
PGR							
Gastric cancer	43	407	79.6%	47.8%	49.9%	9.6%	97.1%
Nongastric cancer	11	373					
G-17 combined with PGII							
Gastric cancer	34	230	63.0%	70.5%	70.0%	12.9%	96.5%
Nongastric cancer	20	550					
G-17 combined with PGR							
Gastric cancer	38	233	70.4%	70.1%	70.1%	14.0%	97.2%
Nongastric cancer	16	547					
PGII combined with PGR							
Gastric cancer	35	309	64.8%	60.4%	60.7%	10.2%	96.1%
Nongastric cancer	19	471					

## Data Availability

The data used to support the findings of this study are available from the corresponding author upon request.
